# Characterization of HIV-1 envelope gp41 genetic diversity and functional domains following perinatal transmission

**DOI:** 10.1186/1742-4690-3-42

**Published:** 2006-07-04

**Authors:** Rajesh Ramakrishnan, Roshni Mehta, Vasudha Sundaravaradan, Tiffany Davis, Nafees Ahmad

**Affiliations:** 1 Department of Microbiology and Immunology, College of Medicine, The University of Arizona Health Sciences Center, Tucson, Arizona 85724, USA; 2 Current Address : Department of Molecular Virology and Microbiology, Baylor College of Medicine, Houston, Texas, 77030, USA

## Abstract

**Background:**

HIV-1 envelope gp41 is a transmembrane protein that promotes fusion of the virus with the plasma membrane of the host cells required for virus entry. In addition, gp41 is an important target for the immune response and development of antiviral and vaccine strategies, especially when targeting the highly variable envelope gp120 has not met with resounding success. Mutations in gp41 may affect HIV-1 entry, replication, pathogenesis, and transmission. We, therefore, characterized the molecular properties of gp41, including genetic diversity, functional motifs, and evolutionary dynamics from five mother-infant pairs following perinatal transmission.

**Results:**

The gp41 open reading frame (ORF) was maintained with a frequency of 84.17% in five mother-infant pairs' sequences following perinatal transmission. There was a low degree of viral heterogeneity and estimates of genetic diversity in gp41 sequences. Both mother and infant gp41 sequences were under positive selection pressure, as determined by ratios of non-synonymous to synonymous substitutions. Phylogenetic analysis of 157 mother-infant gp41 sequences revealed distinct clusters for each mother-infant pair, suggesting that the epidemiologically linked mother-infant pairs were evolutionarily closer to each other as compared with epidemiologically unlinked sequences. The functional domains of gp41, including fusion peptide, heptad repeats, glycosylation sites and lentiviral lytic peptides were mostly conserved in gp41 sequences analyzed in this study. The CTL recognition epitopes and motifs recognized by fusion inhibitors were also conserved in the five mother-infant pairs.

**Conclusion:**

The maintenance of an intact envelope gp41 ORF with conserved functional domains and a low degree of genetic variability as well as positive selection pressure for adaptive evolution following perinatal transmission is consistent with an indispensable role of envelope gp41 in HIV-1 replication and pathogenesis.

## Background

Perinatal transmission occurs at a rate of approximately 30% and accounts for approximately 90% of all HIV-1 infections in children [1]. The infection can occur antepartum (before childbirth; during pregnancy); intrapartum (during childbirth), or postpartum (through breastfeeding). Data from well-performed studies suggest strongly that regimens, including those that substitute oral for intravenous therapy during labor and delivery, can be expected to reduce the risk of vertical transmission of up to 50% [2,3]. However, transmission of antiretroviral therapy (ART) resistant mutants from mother-to-infant has been reported [4]. Genetic analysis of HIV-1 sequences, including *gag *p17 [5], *env *V3 [6], reverse transcriptase [7], *gag *NC [8], *tat *[9], *rev *[10], *vif*[11], *vpr *[12], *vpu *[13] and *nef*[14] from infected mother-infant pairs following perinatal transmission suggest a high conservation of functional domains of these genes and a close relationship between epidemiologically linked mother-infant pairs. In addition, analysis of HIV-1 *env *[15], *vif *and *vpr *[16] and *gag *p17 [17] regions from infected mothers who failed to transmit the virus to their infants in the absence of antiretroviral therapy (non-transmitters) showed a limited heterogeneity of the sequences and low conservation of functional domains. However, other regions of HIV-1 may also play a critical role in transmission and pathogenesis.

One such gene product, gp41, is present on the surface of HIV-1 non-covalently bound to gp120, is responsible for fusion of viral envelope to the plasma membrane of the host cell and is essential for HIV-1 entry and replication. The Env gp41 is comprised of an extraviral domain (ectodomain), a membrane spanning region and an unusually long endodomain within the virus. The ectodomain of gp41 consists of an amino-terminal fusion domain and N- and C-terminal heptad repeats (HR-1 and HR-2, respectively). The gp41 amino terminus is a highly hydrophobic region bearing the FLG motif called fusion peptide (FP), which makes the initial contact with the target membrane and can fuse biological membranes by itself. The two heptad repeat regions self-assemble into a thermostable six-helix bundle, consisting of a trimeric coiled-coil interior (HR-1) with three exterior helices (HR-2) packed in the grooves of the trimer in an antiparallel manner, which represents the fusion-active conformation of gp41 [18].

The endomain of gp41 encodes a Tyr-based motif that interacts with the AP-2 clathrin adaptor protein [19] and is required for optimal viral infectivity [20]. Two lentivirus lytic peptides (LLPs) in this domain which are capable of binding and disturbing lipid bilayers, interact with calmodulin and inhibits Ca^2+^-dependent T-cell activation [21]. There are four sites in gp41 for N-linked glycosylation that promote efficient Env-mediated cell-to-cell fusion [22] but are largely dispensable for viral replication [23]. Although extensive mutational studies have been performed to evaluate the functional domains of gp41 in viral replication, information on the molecular properties of gp41 associated with perinatal transmission and pathogenesis is lacking. Therefore, we have analyzed the gp41 sequences from five HIV-1 infected mother-infant pairs in an effort to understand the molecular properties of gp41 that may be associated with perinatal transmission.

Here we show that the open reading frame of envelope gp41 was highly conserved in the mother-infant pairs' sequences. In addition, there was a low degree of heterogeneity and high conservation of functional domains essential for gp41 activity. These findings may be helpful in the understanding of molecular mechanisms of HIV-1 perinatal transmission and identifying new targets for developing intervention strategies.

## Results

### Phylogenetic analysis of env gp41 sequences from mother-infant pairs

**Figure 1:**
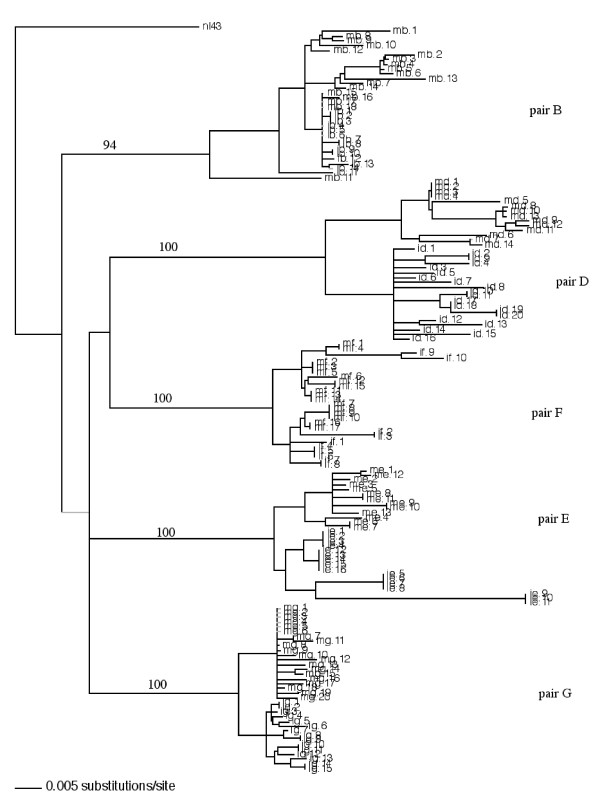
Phylogenetic analysis of 157 envelope gp41 sequences from five mother-infant pairs following perinatal transmission. The neighbor-joining tree is based on the distances calculated between the nucleotide sequences from the five mother-infant pairs. Each terminal node represents one gp41 sequence. The numbers on the branch points indicate the percent occurrences of the branches over 1000 bootstrap resamplings of the data set. The sequences from each mother formed distinct clusters and are well discriminated and in confined subtrees, indicating that variants from the same mother are closer to each other than to other mothers' sequences and that there was no PCR cross contamination. These data were strongly supported by the high bootstrap values indicated on the branch points.

We performed multiple independent polymerase chain reaction (PCR) amplification from peripheral blood mononuclear cells (PBMC) DNA of 5 mother-infant pairs by limit end dilution method. Ten to twenty clones from each patient were obtained and sequenced. Phylogenetic analysis was first performed on the sequences by constructing a neighbor-joining tree of the 157 *env *gp41 sequences from the five mother-infant pairs and the reference strain NL4-3 (subtype-B) as shown in Fig. 1. The neighbor-joining tree was based on the distances calculated between the nucleotide sequences from the five mother-infant pairs and generated by incorporating a best-fit model of evolution into PAUP* [24]. Each terminal node represents one gp41 sequence. The validity of the tree was assessed by bootstrapping the data sets for 1000 times. Phylogenetic reconstructions of the mothers' viral sequences showed distinct clusters corresponding to their respective mother-infant pair and from the NL4-3 control strain, indicating absence of PCR product contamination. The tree also established epidemiological linkage between the transmitting mother and her infant. The mother and infant sequences were generally separated in distinct sub-clusters except for pair B and pair F, where the mother and infant sequences were intermingled. The separation of mother and infant sequences in most pairs indicate that the recipient variant still retained identity to the one or few transmitting variants found in the mothers. The distinct clustering of mother-infant pair sequences and confinement within subtrees also indicate that epidemiologically linked sequences were closer than epidemiologically unlinked sequences. The phylogenetic analysis was strongly supported by high bootstrap values.

### Analysis of coding potential of env gp41 sequences from mother-infant pairs

**Figure 2:**
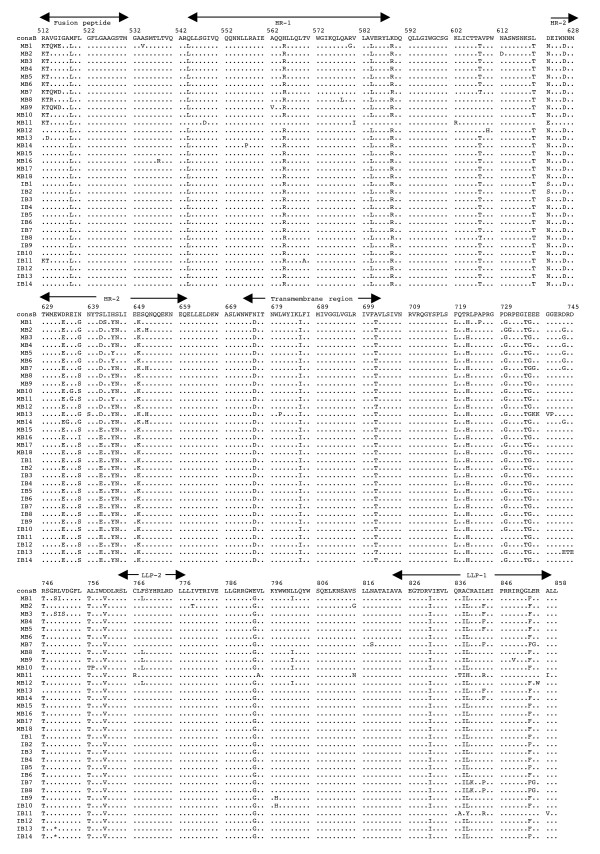
Multiple sequence alignment of the deduced amino acids encoded by envelope gp41 gene of HIV-1 from mother-infant pair B following perinatal transmission. The sequences of pair B are aligned to consensus B (cons B) on top. Each line refers to a clone identified by a clone number preceded by MB (for mother B sequences) and IB (for infant B sequences). Dots indicate amino acid agreement with cons B, dashes represent gaps, and asterisks represent stop codons. The functional motifs of gp41 are indicated above the alignment.

**Figure 3:**
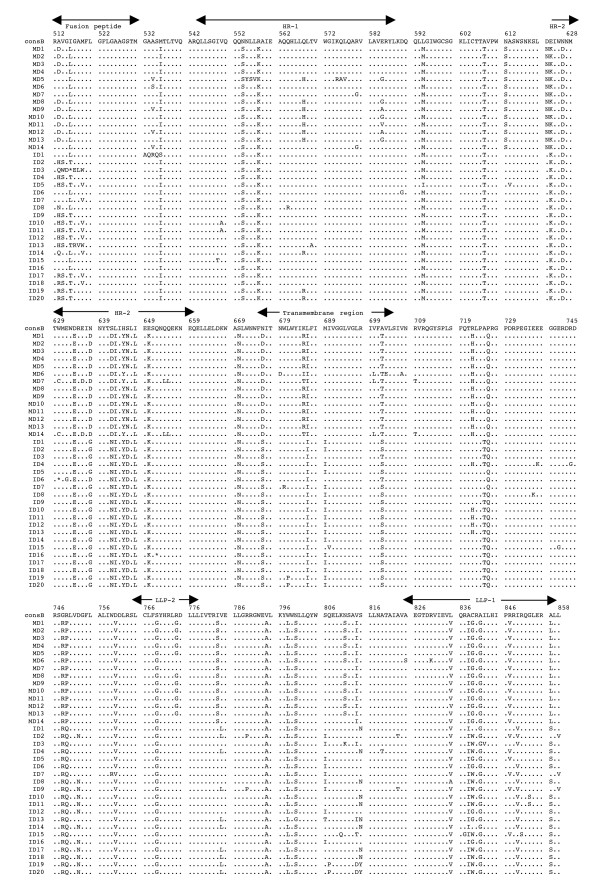
Multiple sequence alignment of the deduced amino acids encoded by envelope gp41 gene of HIV-1 from mother-infant pair D following perinatal transmission. Each line refers to a clone identified by a clone number preceded by MD (for mother D sequences) and ID (for infant D sequences). The sequences of pair D are aligned to consensus B (cons B) on top. Dots indicate amino acid agreement with cons B, dashes represent gaps, and asterisks represent stop codons. The functional motifs of gp41 are indicated above the alignment.

**Figure 4:**
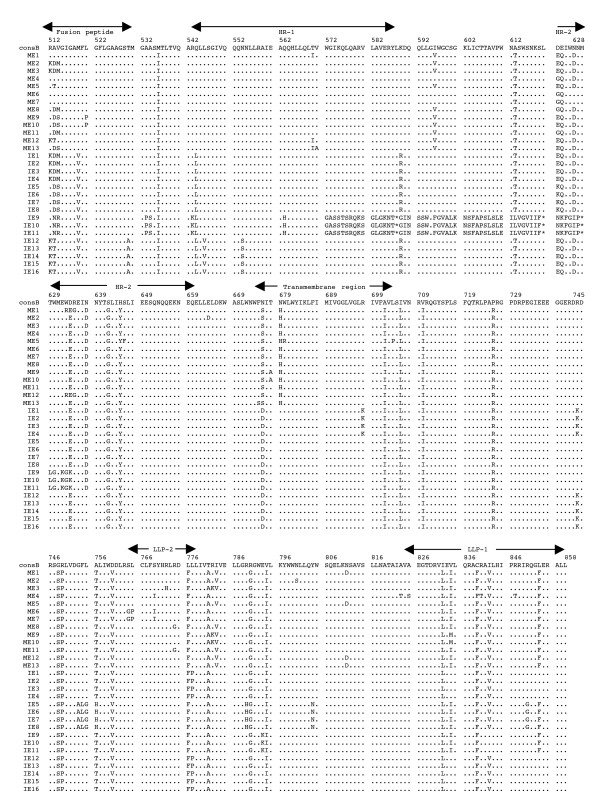
Multiple sequence alignment of the deduced amino acids encoded by envelope gp41 gene of HIV-1 from mother-infant pair E after perinatal transmission. Each line refers to a clone identified by a clone number preceded by ME (for mother E sequences) and IE (for infant E sequences). The sequences of pair E are aligned to consensus B (cons B) on top. Dots indicate amino acid agreement with cons B, dashes represent gaps, and asterisks represent stop codons. The functional motifs of gp41 are indicated above the alignment.

**Figure 5:**
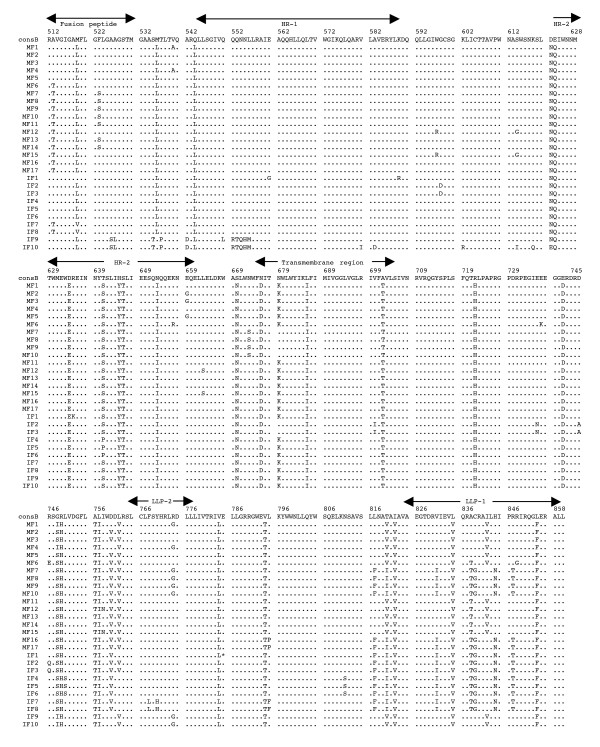
Multiple sequence alignment of the deduced amino acids encoded by envelope gp41 gene of HIV-1 from mother-infant pair F after perinatal transmission. Each line refers to a clone identified by a clone number preceded by MF (for mother F sequences) and IF (for infant F sequences). The sequences of pair F are aligned to consensus B (cons B) on top. Dots indicate amino acid agreement with cons B, dashes represent gaps, and asterisks represent stop codons. The functional motifs of gp41 are indicated above the alignment.

**Figure 6:**
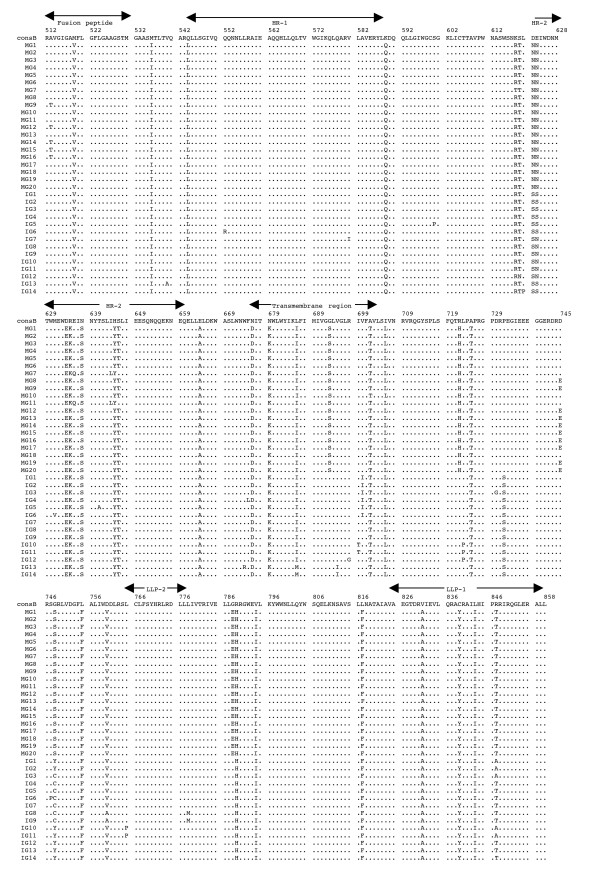
Multiple sequence alignment of the deduced amino acids encoded by envelope gp41 gene of HIV-1 from mother-infant pair G after perinatal transmission. Each line refers to a clone identified by a clone number preceded by MG (for mother G sequences) and IG (for infant G sequences). The sequences of pair G are aligned to consensus B (cons B) on top. Dots indicate amino acid agreement with cons B, dashes represent gaps, and asterisks represent stop codons. The functional motifs of gp41 are indicated above the alignment.

The multiple alignments of the deduced amino acid sequences of HIV-1 *env *gp41 gene isolated from the PBMC DNA from the five mother-infant pairs following perinatal transmission is shown in Figs. 2 to 6. The alignment was done in reference to HIV-1 consensus B (consB) sequence. In the alignment, the top sequence is reference consensus B sequence and pairs B, D, E, F, and G represent the five mother-infant pairs. M indicates mother sequences and I indicate infant sequences. Dots represent amino acids identical to consB sequence, dashes indicate gaps, substitutions are shown by single letter codes for the changed amino acid and asterisks represent stop codons. Of the 157-gp41 clones analyzed, 133 contained intact gp41 open reading frames, which correlate to 84.17% frequency of intact open reading frames. The mothers and infants sets showed frequencies of 82.93% and 86.67% intact open reading frames, respectively. We found that 9 clones had one or more stop codons. The gp41 sequences were derived from PBMC DNA that represents both replicating and non-replicating forms of proviral DNA. It is noteworthy that each mother-infant pair gp41 sequences displayed pair-specific amino acid patterns that were not seen in epidemiologically unlinked pairs. In addition, there were several common signature motifs seen in all mother-infant pairs' sequences, including Asp634→Glu, His645→Tyr and Asn676→Asp.

### Variability of env gp41 sequences of epidemiologically linked mother-infant pairs

The degree of genetic variability of the *env *gp41 sequences from five mother-infant pairs was determined on the basis of pairwise comparison of the nucleotide and deduced amino acid sequences. The minimum, median and maximum nucleotide and deduced amino acid distances are shown in Table 2. The nucleotide distances ranged between 0% and 5.2% with a median of 0.97% for mothers, 0% to 4.8% with a median of 1.26% for infants. The amino acid distances ranged from 0% to 5.96% with a median of 1.16% for mothers and from 0% to 6.89% with a median of 2.04% for infants. The nucleotide and amino acid distances of gp41 sequences between epidemiologically unlinked individuals were also determined. Epidemiologically unlinked individuals had a median nucleotide distance of 9.01% with a maximum of 15.91% and a median amino acid distance of 13.65% with a maximum distance of 40.2%, respectively. These distances are significantly higher than epidemiologically linked mother-infant pairs, which ranged from 0% to 6.07% with a median of 1.85% (nucleotides) and 0% to 6.89% with a median of 3.23% (amino acids). Some of the hypermutated and severely defective clones were not included in the distance calculation. These sequences are frequently seen in *pol *and *env *regions of HIV-1 genome [25] and inclusion of these clones gives an incorrect picture of viral heterogeneity. We also investigated if the low variability of gp41 sequences seen in our mother-infant pair isolates was due to errors made by TaKaRa LA *Taq *polymerase used in this study. We did not find any errors when a known HIV-1 *env *gp41 sequence from NL4-3 was used for PCR amplifications and DNA sequencing using TaKaRa LA *Taq *polymerase.

**Table 1:** Patient demographic, clinical, and laboratory parameters of HIV-1 infected mother-infant pairs.

Patient	Age	Sex	CD4+ cells/mm3	Length of infection^b^	Antiviral drug	Clinical evaluation^c^
**Mothers**						

B	28 yr		509	11 mo	None	Asymptomatic
D	31 yr		480	2 yr 6 mo	None	Asymptomatic
E	26 yr		395	2 yr	ZDV^d^	Symptomatic AIDS
F	23 yr		692	2 yr 10 mo	None	Asymptomatic
G	23 yr		480	10 mo	None	Asymptomatic

**Infants**						

B	4.75 mo	M	1942	4.75 mo	None	Asymptomatic, P-1A
D	28 mo	M	46	28 mo	ddC^e^	Symptomatic AIDS, P-2A, B, F, failed ZDV therapy
E	34 mo	M	588	34 mo	ZDV^d^	Symptomatic AIDS, P-2A
F	1 wk	M	2953	1 wk	ZDV^d^	Asymptomatic, P-1A
G	24 mo	F	4379	24 mo	ZDV^d^	Asymptomatic, P-1B

^b ^Length of infection: The closest time of infection that we could document was the first positive HIV-1 serology date or the first visit of the patient to the AIDS treatment center where all the HIV-1 positive patients were referred to as soon as an HIV-1 test was positive. As a result, these dates may not reflect the exact dates of infection.

Mother and infant samples for each pair were collected at the same time.

^c ^Evaluation for infants is based on CDC criteria [65]

^d^ZDV: Zidovudine

^e^ddC: Zalcitabine

**Table 2:** Distances in the envelope gp41 sequences within mother sets, within infant sets and between mother-infant pair sets

Patient	Within mothers			Within infants			Between mother-infants		
**Nucleotides**									

	Min	Median	Max	Min	Median	Max	Min	Median	Max

B	0	1.86	5.2	0	0.38	2.13	0	1.76	5.2
D	0	1.72	3.99	0	2.05	3.65	0	1.82	3.99
E	0	1.36	2.65	0	2.55	4.8	0	2.65	6.07
F	0	1.17	2.05	0	1.31	2.35	0	1.66	2.35
G	0	0.56	1.23	0	0.97	1.46	0	1.44	2.78
Total	0	0.97	5.2	0	1.26	4.8	0	1.85	6.07

**Ammo acids**									

	Min	Median	Max	Min	Median	Max	Min	Median	Max

B	0	2.34	5.04	0	0.58	4.44	0	2.04	5.04
D	0	0.87	5.96	0	3.53	6.89	0	3.23	6.89
E	0	2.04	4.44	0	2.04	5.66	0	2.64	5.66
F	0	2.34	4.13	0	2.03	3.53	0	2.34	4.13
G	0	0.29	1.75	0	1.75	2.94	0	2.19	4.44
Total	0	1.16	5.96	0	2.04	6.89	0	3.23	6.89

Distances are expressed as percent nucleotides (for nucleotide sequences) or percent amino acids (for amino acid sequences). Totals were calculated for all pairs taken together.

### Dynamics of env gp41 sequence evolution in mother-infant pairs

We next examined the population genetic parameters using the Watterson model and the program COALESCE assuming a constant population size using a Kimura two-parameter model of sequence evolution [26,27]. The genealogical structure of a sample from a population contains information about that population's history. The mathematical theory relating a genealogy to the structure of its underlying population is called coalescent theory. The genetic diversity parameter, θ, estimated as nucleotide substitutions per site per generation for each patient's HIV-1 population is shown in Table 3. The levels of genetic diversity among mother sets, as estimated by Watterson and Coalesce methods, ranged from 0.01 to 0.02 and 0.01 to 0.03, respectively. Among infant sets, the levels of genetic diversity ranged from 0.01 to 0.03 when estimated by both Watterson and Coalesce methods. The HIV-1 populations found in the mothers displayed overall same genetic diversity (0.02) when compared to HIV-1 populations found in the infants (0.02).

**Table 3:** Estimates of genetic diversity of envelope gp41 sequences within mother sets and within infant sets

**Patient**	**Mother sets**			**Infant sets**		
	N	θ_w_	θ_c_	N	θ_w_	θ_c_

B	14	0.02	0.03	14	0.01	0.01
D	14	0.02	0.03	20	0.03	0.03
E	13	0.02	0.03	13	0.01	0.01
F	17	0.01	0.01	8	0.01	0.01
G	20	0.01	0.02	15	0.01	0.02
**Total**	**78**	**0.02**	**0.02**	**70**	**0.01**	**0.02**

N = Number of gp41 clones sequenced

θ_w _= genetic diversity estimated by the method of Watterson

θ_c _= genetic diversity estimated by Coalesce

Totals were calculated as averages of all values

### Rates of accumulation of non-synonymous and synonymous substitutions

Natural selection is assumed to operate mainly at the amino acid sequence level because most of the important biological functions in the organisms seem to be performed mainly by proteins. The rate of synonymous substitutions (dS) may be more or less similar to mutation rate, whereas the rate of nonsynonymous substitutions (dN) may vary according to the type and strength of natural selection. If positive selection occurs, dN will be expected to be faster than dS and the opposite can be expected in case of negative selection. Although several methods have been proposed to calculate the rate of dN and dS, these models assume that all sites in the sequence are under the same selection pressure. It is likely that since different sites in a protein have varying functional and structural roles, the selection pressure acting on them might not be uniform. We have used a maximum likelihood model modified by Nielson and Yang [28] to analyze evolutionary processes acting on *env *gp41 gene, considering the codon instead of the nucleotide as unit of evolution. The viral population in all the patient pairs studied showed a dN/dS ratio of more than 1, which is indicative of positive selection pressure (Table 4). Interestingly, the viral population in infants consistently showed more selection pressure than their respective mothers indicating that adaptive evolution in these patients was probably influenced not only by the immune system but also the fact that most of these infants were under antiretroviral therapy (Table 1).

**Table 4:** dN/dS ratios in the envelope gp41 sequences within mother sets and within infant sets.

	**Mother sets**						**Infant sets**				
Pair	N	P1	P2	P3	dN/dS	Pair	N	P1	P2	P3	dN/dS

B	14	85.24	0.0	14.76	6.04	B	14	69.66	21.07	9.27	13.02
D	14	78.06	20.28	1.65	7.88	D	20	55.33	40.10	4.57	21.97
E	13	55.72	0	44.27	6.77	E	13	15.73	70.44	13.83	5.36
F	17	0	96.2	3.7	1.93	F	8	65.53	0.0	34.47	3.79
G	20	95.71	3.29	1.00	3.85	G	15	0	99.58	0.41	16.21
**Total**	**78**	**62.95**	**23.95**	**13.08**	**5.29**	**Total**	**70**	**41.25**	**53.13**	**12.51**	**12.07**

N = Number of gp41 clones sequenced; P1 = proportion of conserved codons as a percent; P2 = proportion of neutral codons as a percent; P3 = proportion of positively selected codons as a percent; dN/dS = dN/dS ratio at P3 sites. Totals were calculated as the average of all values.

### Analysis of functional domains of env gp41 in mother-infant isolates

The domain structure of gp41 can be divided into an ectodomain, a membrane spanning region and an endodomain. The N-terminus of the ectodomain is a highly hydrophobic region called the fusion peptide (FP), which makes the initial contact of the glycoprotein with the target membrane and can fuse the virus to the plasma membrane. Mutations including Val513→Glu, Leu520→Arg, Ala526→Glu, Leu538→Arg, Gln541→Leu have been shown to completely abolish syncytium-inducing ability and production of infectious virus [29]. Examination of the five mother-infant pairs' gp41 sequences in our study showed a change of Val513→Gln (some clones of pair B) (Fig. 2), Val513→Ser (some clones of infant D) (Fig. 3), and Val 513→Met (some clones of pair E) (fig. 4). All the other critical residues in this important motif were highly conserved. While non-conservative mutations in the leucine/isoleucine backbone Ile574→Asp of HR1 abrogate viral infectivity; expression, oligomerization, and localization of the fusion protein complexes remains unaffected [30-32]. None of the sequences analyzed harbored Ile574→Asp mutation. Mutational studies have revealed other changes that can affect fusion activity including Val571→Glu, and Gln576→Glu [33]. All the clones analyzed here showed conservation of the above-described residues, although some changes were observed in the flanking regions (Figs. 2 to 6). The changes in HR1 motif include Asn554→Ser and Arg558→Lys in (pair D), Gln544→Leu (pair B, infant E, pairs F and G), His565→Arg and Lys589→Arg (pair B), Lys589→Gln (pair G). The changes in HR2 motif include Asn625→Asp (all pairs except F), Asp633→Glu (all pairs), Asn637→Gly/Ser (pair B, D and G).

It has been shown that N-linked glycosylation can serve to modulate the exposure of HIV-1 proteins to immune surveillance in patients [34,35]. There are three to four N-glycan attachment sites (residues 612–642) in the C-terminal half of the ectodomain. We examined our sequences for substitutions in these glycosylation sites and found that there was a relatively high degree of conservation, except for the following changes: mother D (Asn612→Ser), pair E (Ala613→Thr), pair G (Lys618→Arg and Ser619→Thr), pair F (Thr640→Ser and Thr640→Pro). A recent report noted the presence of Ala613→Thr change in *env *genes derived from brains of AIDS patients [36].

Next the membrane-spanning domain (MSD) of gp41 that anchors Env on the lipid bilayer was examined. Several studies have indicated that the MSD is involved in membrane fusion as the glycosylphosphatidylinositol-anchored Env of HIV-1, which lacks the MSD and endodomain could not induce syncytia [37,38]. A recent report [39] found that the well-conserved glycine residues that form the GXXXG motif (where G, glycine; X, any amino acid) found at the helix-helix interface of MSD α-helix, tolerated mutations without affecting fusion function. The mother-infant sequences in this study showed a high conservation of the GXXXG motif except in all clones of mother G where there was a Gly693→Ser change (Fig. 6).

Further, important motifs in the endodomain of gp41 were analyzed. A dileucine (amino acids 856–857) motif critical for association with AP-1 clathrin adaptor and transport of Env precursors through the trans-Golgi network to the cell surface is found at the C-terminal end of gp41 [40]. This dileucine motif is preceded by an acidic amino acid (Glu 853) which is thought to expose the motif allowing it to be recognized by adapters [41]. The sequences from five HIV-1 infected mother-infant pairs show a high degree of conservation in both the dileucine motif and the preceding acidic amino acid (Glu 853) indicating that Env trafficking in these clones were not affected. Piller *et al*. [42] performed a comprehensive mutational analysis of the conserved domains in the endodomain and found that there was a pronounced reduction of glycoprotein incorporation upon deletion of the conserved region of P(846)RRIR(850) or replacement of the central RR with KK. While our mother-infant pairs' sequences did not have any of these changes, several substitutions including Arg846→Val (pair D), and Arg846→Thr (pair E and G) were seen. The α-helical motifs of LLP-1 and LLP-2 have been implicated in virus-mediated cytopathicity by binding to calmodulin, a critical mediator of cytoplasmic signal transduction cascades in T-cells [43]. Upon analyzing the five mother-infant pairs' sequences (Figs. 2 to 6), changes were observed in these motifs including, Ser768→Gly (pair D), Leu775→Phe and Thr780→Ala (pair E), Val783→Leu and Ala837→Thr (pair F), Ala836→Arg (all pairs), Ala837→Ile in pairs B and D.

### Analysis of motifs targeted by HIV-1 fusion inhibitors in mother-infant gp41 sequences

Although none of our patients were on fusion inhibitor (T-20), we analyzed the target motifs for T-20 as well as naturally occurring T20 resistant mutants. T20 (enfuvirtide), a 36-mer synthetic peptide fusion inhibitor, corresponding to the overlapping regions within HR2 [44] exerts its antiviral activity by interacting with a target sequence in HR1 that inhibits association with native HR2. The T20 sensitive region lies between positions 548–550 (amino acids Gly, Ile, Val) and selection of resistant viruses have been found during serial *in vitro *passages in the presence of T20 [45]. Examination of all our mother-infant clones showed a sensitive motif to T20, except two clones in infant D (ID) that have a Val550→Ala (ID 10, 11) or Ile549→Thr (ID15) (Fig. 2). Poveda *et al *[46], studied the evolution of genotypic and phenotypic resistance to T20 in HIV-infected patients and found that there was a clear relationship between selection of single changes at the GIVQQQNNLL motif and loss of susceptibility to T20. Some clones in infant E (IE12-16) had an Asn554→Ser and two clones in infant F (IF9 and 10) an Asn554→Gln change due to a frame shift, indicating that these patients might be resistant to T20 treatment. A Leu556→Met variation present in the HR-1 domain has been associated with the development of clinical resistance but is generally found in combination with additional changes [47]. Of all the 157 clones we analyzed, only two clones in the present study (IF9 and IF10) had a Leu556→Met change. A recent study found that NeoR6, an aminoglycoside-arginine conjugate, inhibits HIV-1 replication by interfering with the fusion step and that the resistant isolates had a Ser669→Arg and a Phe671→Tyr change in the HR2 region [48]. Most of our gp41 sequences showed no change, except for pairs D and F sequences that had a Ser669→Asn substitution.

Human monoclonal anti-gp41 antibodies, 2F5 and 4E10, are known to prevent membrane fusion and neutralizes a broad range of HIV-1 primary isolates [49,50]. The 2F5 epitope on gp41 includes the sequence ELDKWA, with the core residues, DKW, being critical for antibody binding while 4E10 targets the adjacent WFNI sequence. A high degree of conservation of these epitopes was found in the five mother-infant pairs' sequences, except pair G that had a Glu663→Ala change, suggesting susceptibility of these clones to neutralization by 2F5 and 4E10.

### Analysis of immunologically relevant mutations in the CTL epitopes of gp41 sequences

Evasion of the host cytotoxic T-lymphocytes (CTL) response through mutation of key epitopes is a major challenge for both natural and vaccine-induced immune control of HIV-1. Immunodominant responses generally are effective but HIV-1 generates variants that expose CTL to a large pool of mutants impairing immune responsiveness [51]. Escape from CTL control is indicated by a mutation that occurs in the T-cell epitope and becomes fixed in the virus population, resulting in an *in vivo *competitive advantage for the virus with reduction of the T-cell response to the wild-type epitope. Escape mutants can arise early or late in HIV-1 infection [51,52], and can also be transmitted [53]. Geels *et al *[54] have described two epitope clusters (residues 770–780 and 835–843) in HIV-1 infected patients over a period of 80 months that showed non-fixation of mutations. In the epitope encompassing residues 770–780, variant residues (Ile777→Val and Val778→Ile) were seen late (49 months) in infection, whereas in the other epitope (835–843), Ala836→Thr and Tyr838→Phe occurred early in infection. In the clones that were analyzed from five HIV-1 infected mother-infant pairs, Ile777 and Val778 were conserved across the board. However, several changes were observed, including a Cys838→Leu (pair B), a Cys838→Gly/Trp (pair D), a Cys838→Phe (pair E), or Cys838→Gly (pair F). These changes in the mother and infant clones suggest that these escape variants evolved to escape immune responses and influence transmission. In another study, infected mothers were found to transmit HIV-1 to their infants despite a strong CTL response to epitope 557–565 (RAIEAQQHL), suggesting generation of escape variants in the mothers [55]. Mother-infant sequences in the current investigation showed a high conservation of this epitope with few substitutions in pair B (His564→Arg) and in pair D (Arg557→Lys).

## Discussion

We provide evidence that a high frequency of intact envelope gp41 ORF and functional domains required for gp41 activity were maintained in five mother-infant pairs following perinatal transmission. In addition, there was a low degree of viral heterogeneity and estimates of genetic diversity and a positive selection pressure on gp41 sequences from mother-infant pairs. Although gp41 plays an important role in viral replication and pathogenesis, no systematic study has been performed that analyzed the functional domains, evolutionary dynamics and the effect of adaptive evolution of gp41 involved in perinatal transmission. As such, our analysis of gp41 sequences on five mother-infant pairs support the notion that gp41 plays an important role in HIV-1 infection, replication and pathogenesis in infected mothers and their perinatally infected infants.

In the analysis of gp41 sequences from five mother-infant pairs, we found the maintenance of intact gp41 open reading frames with a high frequency in mothers, of 82.93%, and in infants, of 86.67%, following perinatal transmission. This is comparable with other regions of HIV-1 genome from same mother-infant pairs, including *gag *p17MA (86.2%), reverse transcriptase (87.2%), *vif *(89.8%), *vpr *(92.1%), *vpu *(90.3%), *nef *(86.7%), *tat *(90.9%) and *rev *(96.6%) [5,7,9-14]. The maintenance of gp41 ORF confirms the importance of this protein in the viral life cycle and its role in perinatal transmission of HIV-1. The phylogenetic analysis showed distinct clusters corresponding to their respective mother-infant pair and from the NL4-3 control strain, indicating absence of PCR product contamination as shown in Figure 1. This analysis was supported by high bootstrap values. The separation of mother and infant sequences in most pairs indicate that the recipient variant still retained identity to the one or few transmitting variants found in the mothers. Similar conclusions were reached upon analysis of *env *gp120 V3 region [6]. Moreover, this distinct clustering of mother-infant pair sequences and confinement within subtrees also indicate that epidemiologically linked sequences were closer than epidemiologically unlinked sequences.

Analysis of genetic variability, measured as nucleotide and amino acid distances, showed a low degree of variability in most mother-infant pairs (Table 2) and are comparable to sequence distances for other conserved genes such as *gag *[5], reverse transcriptase [7], *vif*[11], and *vpr *[12], but lower than *env *gp120 V3 [6], *vpu *[13] and *rev *[10] from the same mother-infant pairs. Similar results were obtained for the estimates of genetic diversity. Consistent with the critical role of gp41 in HIV-1 pathogenesis [56], it was found that there was a positive selection pressure (dN/dS) on the gp41 sequences to change (Table 4) but maintain the functional motifs (Figs. 2, 3, 4, 5, 6). These values are comparable to other HIV-1 genes from infected patients, including *env *gp120 V3, *gag*, reverse transcriptase, *tat, vif *and *vpr *[5-7,9,11,12]. Further analysis of the dN/dS values show that the viral population in infants experienced more selective pressure than their respective mothers, suggesting that adaptive evolution in these infants was probably influenced both by the host immune response and by antiretroviral therapy. However, a low degree of genetic diversity and the increased selection pressure could be indicative of the fact that the virus was rapidly evolving to a more stable variant.

The functional domains and motifs required for gp41 activity in the deduced amino acid sequences, including hydrophobic fusion peptide (FP), HR1, HR2, precursor (gp160) cleavage, and cell signaling were mostly conserved in our mother-infant pairs sequences. Mutations in the FLG motif of FP region that completely abolished syncytium-inducing ability and infectious virus production [29] were found to be highly conserved in most of our mother-infant pairs' sequences. In addition, none of our gp41 sequences harbored mutations in the leucine/isoleucine backbone of HR region which have been shown to affect viral infectivity [18]. Furthermore, no other mutations or substitutions were found in our mother-infant gp41 sequences that may affect (i) hydrophobic cavities in the HR1 region, (ii) membrane fusion activity by destabilizing the trimer of hairpin structures, (iii) packing interactions between the amino and carboxy terminal helices of gp41, and (iv) the structural integrity of the trimer of hairpins [57].

HIV-1 gp41 typically contains three or four sites for N-glycan (N-X-S/T) attachment located at the C-terminal of the ectodomain, which may serve to modulate the exposure of HIV-1 proteins to immune surveillance in patients [34,35]. We found that there was a relatively a high degree of conservation of the four-glycosylation sites in the sequences analyzed with some changes in few sequences. Interestingly, a substitution of Ala613→Thr was seen in our gp41 sequences (pair E) that has been previously shown to be derived from brain of patients with AIDS [36]. This suggests that the variants in patient E have the potential to be neurotropic and may cause CNS disorders, which are commonly seen in infected infants [58]. In the other three-glycosylation sites some changes were seen that do not affect the consensus motif. The membrane spanning domain (MSD) that is critical for functional integrity [39] was highly conserved in our gp41 sequences, except in all sequences of mother G (Gly690→Ser). As serine residues also have a stabilizing effect on the helix structure, it is possible that this substitution observed in mother G sequences might not compromise the functional activity. The endodomain encodes two lentivirus lytic peptides (LLPs) which inhibit Ca^2+^-dependent T-cell activation [21] and a leucine zipper motif between LLP-1 and LLP-2 that plays an important role in HIV-1 replication and pathogenesis [59]. The gp41 sequences analyzed in our study showed a high degree of conservation of essential residues in this leucine zipper motif.

With respect to T20 target motifs, most of the clones analyzed in this study showed a high degree of conservation, which was expected because these patients were never treated with T20 inhibitors. In pair D sequences, four sequences in infant E (Asn554→Ser), and two sequences in infant F (Asn554→Gln) substitutions were found, suggesting that mutants naturally occurred in these patients and might be resistant to T20. A wide range of susceptibility to T-20 has been described in fusion peptide naive virus isolates from patients [60]. Recent studies [61,62] have concluded that the susceptibility to T20 was influenced by coreceptor usage but not by polymorphisms in the gp41 N helix. These studies demonstrated that viruses containing a CCR5-utilizing V3 loop were four- to eightfold less susceptible to T20 than the CXCR4-utilizing parent strains. We and others have shown that CCR5 utilizing macrophage-tropic (R5) viruses are transmitted from mother to infant [63,64], suggesting that T20 might help reduce viral load and may prevent perinatal transmission.

Evasion of the CTL and neutralizing antibody responses through mutation of key epitopes is a major challenge for both natural and vaccine-induced immune control of HIV-1 [55]. CTL escape mutants can arise early or late in HIV-1 infection [51,52], and can also be transmitted [53]. Although CTL are HLA restricted, we analyzed several CTL epitopes recognized by different HLA types in our gp41 sequences. Several substitutions were seen in our gp41 sequences and some of these mutations in the terminal residues could affect peptide processing. Several substitutions were seen in the flanking regions of both functional domains and CTL epitopes. While the relevance of these changes is not clear at this time, biological studies using the gp41 clones obtained in this study could give a better picture of their effects. It would also be interesting to characterize the gp41 region in HIV-1 infected mothers who failed to transmit the virus to their infants in the absence of therapy and perform a comparative study.

Although perinatal transmission of HIV-1 is a multifactorial processes, the results presented in this study not only underscores the importance of gp41 in HIV-1 perinatal transmission but also provides an understanding of the functional and immunological motifs that can be used to develop therapeutic interventions in blocking virus entry.

## Conclusion

We have demonstrated that an intact and functional envelope gp41 gene was maintained in infected mother-infant pairs following perinatal transmission. In addition, there was a low degree of viral heterogeneity and estimates of genetic diversity in epidemiologically linked mother-infant pairs as compared to epidemiologically unlinked individuals. Several amino acid motifs were found as a signature sequences in each mother-infant pair. We also found that the functional motifs of envelope gp41 responsible for fusion, gp160 processing and cytopathogenicity were highly conserved in mother-infant sequences. These findings support the notion that envelope gp41 is essential for HIV-1 infection and pathogenesis in mothers and their perinatally infected infants.

## Methods

### Patient population and sample collection

Blood samples were collected from five HIV-1 infected mothers and their respective vertically infected infants between 1990 and 1995. The children of these mothers were evaluated and found to be infected by repeated testing following guidelines published by the Centers for Disease Control and Prevention [65]. The demographic, clinical and laboratory findings of the HIV-1 infected study subjects are summarized in Table 1. The Human Subjects Committee of the University of Arizona and the Institutional Review Board of the Children's Hospital medical Center, Cincinnati, Ohio, USA, approved this study, and written consent was obtained from the participants of the study (mothers for their infants).

### PCR amplification, cloning, and nucleotide sequencing

DNA was isolated from uncultured PBMC of HIV-1 infected individuals according to a modified procedure described before [6]. The HIV-1 *env *gp41 gene from infected patients' PBMC DNA was amplified using the following primers: Gp41-6 (+) (AGTAAAAATTGAACCATT AGGAGTAGCA, 7678 to 7705, sense), Gp41-7 (-) (CTTTCCCTTACAGCAGGCCATCCAATCAC, 8815 to 8836, anti-sense) as outer primers, and Gp41-8 (+) (CAAGGCAAAGAGAAGAGTGGTT-GCA, 7711 to 7734, sense), Gp41-9 (-) (TACTTTTTGACCACTTGCCACCCAT, 8786 to 8811, anti-sense) as inner primers based on NL4-3 sequence [66]. An equal amount of HIV-1 PBMC DNA (25 to 50 copies, minimum) was used from each patient as determined by end-point dilution and multiple (6 to 8) independent PCRs were performed to obtain clones that were then sequenced and analyzed. PCRs were performed according to the modified procedure described by Ahmad *et al*. [6] using 2.5 U of TaKaRa LA Taq polymerase (Chemicon International) in accordance to manufacturer's protocol. The first reaction was carried out at 94°C for 30 s, 50°C for 45 s and 72°C for 1 min for 35 cycles, with 8 minutes of additional polymerization time in the last cycle. After the first round of PCR, 4 to 8 μl of the above- described amplified product was used for nested PCR, using the inner primers and the same concentrations of other ingredients at 94°C for 30 s, 55°C for 45 s and 72°C for 1 min for 35 cycles, with 8 min of additional polymerization time in the last cycle. PCR was also performed on HIV-1 NL4-3, of which the sequence is known (GenBank accession number M19921) [66], to assess any errors made by the TaKaRa LA Taq polymerase. The PCR products were directly cloned in the pCR2.1 TOPO TA cloning vector, version K2 (Invitrogen). Bacterial colonies were screened for the presence of inserts by restriction enzyme digestion of recombinant plasmid DNA. The positive clones were selected and propagated for DNA isolation followed by nucleotide sequencing of 10 to 20 clones from each patient. Sequencing was performed using the Thermosequenase Cycle sequencing protocol (USB) using Gp41-8 (+) primer. DNA from clones which were positive for gp41 when compared with NL4-3 reference sequence were sent to the core facility (University of Arizona) for sequencing on an ABI PRISM ^® ^370 DNA automated sequencing system (Applied Biosystems). As the reliability of the sequencing was only up to 600 bases, we designed a primer Gp41-5 (+) (5' CAGACCCACCTCCCAATCCCGAGGGGA 3', 8366 to 8392, sense) overlapping the region around 550 bases to walk the entire gp41 sequence. The two sets of sequences were manually joined to generate the complete full-length sequences of HIV-1 gp41 clones. These full-length clones were then used to perform sequence analysis. The sequences were handled with the Wisconsin package, version 10.1 (Genetics Computer Group).

### Sequence analysis

The nucleotide sequences of the gp41 clones were aligned using Clustal X [67] adjusted by hand and then translated into corresponding amino acid sequences. A model of evolution was optimized for the entire nucleotide sequence data set using the Huelsenbeck and Crandall approach [68]. Likelihood scores for different models of evolution were calculated using PAUP*, and a chi square (χ^2^) test was performed by Modeltest 3.06 [69]. The model of choice was incorporated into PAUP* to estimate a neighbor-joining tree. Bootstrap values were based on 1000 neighbor-joining searches. The tree was generated for the nucleotide sequences from the six mother-infant pairs, and the reference HIV-1 sequence, NL4-3, was used as an out-group for the tree display (Fig. 1). Using Modeltest and the Akaike Information Criterion [70], all the null hypotheses were rejected except likelihood settings from best-fit model, transversion model with invariable sites and gamma distribution (TVM+I+G) selected by AIC in Modeltest Version 3.06. The base frequencies were as follows: freq A = 0.3073, freq C = 0.1940, freq G = 0.2653, freq T = 0.2334. The six rate categories were as follows: R (A-C) = 1.4514, R (A-G) = 4.5534, R (A-T) = 0.8671, R (C-G) = 1.3081, R (C-T) = 4.5534, R (G-T) = 1.0. The proportion of invariable sites (I) was 0.1561. The rate heterogeneity was taken into account using a gamma distribution with a shape parameter (a) of the distribution estimated from the data via maximum likelihood. The gamma distribution shape parameter had a value of a = 0.7961 for gp41. Similarly, a model of evolution was optimized for the data set from each pair. These models were used to estimate corrected pairwise nucleotide distances for the data sets from each pair using PAUP* [24]. Mean character amino acid distances were also determined using the Jukes-Cantor model in the Wisconsin Package version 10.1 of GCG. The minimum, maximum and median nucleotide and amino acid distances were calculated for each patient as well as for linked and unlinked patient pairs. The dynamics of HIV-1 evolution was assessed using techniques of population genetics. The genealogical structure of a sample from a population contains information about that population's history. The mathematical theory relating a genealogy to the structure of its underlying population is called coalescent theory [27]. The distribution of coalescence times, that is the times at which two of the sampled individuals have a common ancestor, depends on the effective population size. In population genetics, genetic diversity is defined as θ = 2N_eμ_, where N_e _is the effective population size and μ is the per nucleotide mutation rate per generation. The differences in genetic diversity was examined using the Watterson estimate based on segregating sites and Kuhner estimate assuming variable population size, using the program Coalesce which is part of the Lamarc software package [26,71]. To analyze the evolutionary processes acting upon the gp41 gene, we estimated the ratio of nonsynonymous (dN) to synonymous (dS) substitutions by a maximum likelihood model using codeML, which is part of the PAML package [72]. The Nielsen and Yang model [28] considers the codon instead of the nucleotide as the unit of evolution and thus incorporates three distinct categories of sites. The first category represents the sites that are invariable or conserved (p1, dN/dS = 0); the second category represents sites that are neutral (p2, dN/dS = 1), at which dN and dS are fixed at the same rate; and the third category represents sites that are under positive selection, where dn has a higher fixation rate than ds (p3, dN/dS > 1). The dN/dS was estimated for each patient using both neutral and positive selection models in codeML.

### Nucleotide sequence accession numbers

The sequences have been submitted to GenBank with accession numbers AY880686-AY880842.

## Competing interests

The author(s) declare that they have no competing interests.

## Authors' contributions

RR, RM and TD carried out the PCR, cloning, and sequencing. RR, VS, and NA performed the sequence analysis by computer programs. RR and NA participated in the experimental design, data interpretation, and writing of the manuscript. All authors read and approved the final manuscript.

## References

[B1] Ahmad N (2005). The vertical transmission of human immunodeficiency virus type 1: molecular and biological properties of the virus. Crit Rev Clin Lab Sci.

[B2] Abrams EJ, Wiener J, Carter R, Kuhn L, Palumbo P, Nesheim S, Lee F, Vink P, Bulterys M (2003). Maternal health factors and early pediatric antiretroviral therapy influence the rate of perinatal HIV-1 disease progression in children. Aids.

[B3] Connor EM, Sperling RS, Gelber R, Kiselev P, Scott G, O'Sullivan MJ, VanDyke R, Bey M, Shearer W, Jacobson RL, Jimenez E, O'Neill E, Bazin B, Delfraissy J-F, Culnane M, Coombs R, Elkins M, Moye J, Stratton P, Balsley J (1994). Reduction of maternal-infant transmission of human immunodeficiency virus type 1 with zidovudine treatment. Pediatric AIDS Clinical Trials Group Protocol 076 Study Group. N Engl J Med.

[B4] Frenkel LM, Wagner LE, Demeter LM, Dewhurst S, Coombs RW, Murante BL, Reichman RC (1995). Effects of zidovudine use during pregnancy on resistance and vertical transmission of human immunodeficiency virus type 1. Clin Infect Dis.

[B5] Hahn T, Matala E, Chappey C, Ahmad N (1999). Characterization of mother-infant HIV type 1 gag p17 sequences associated with perinatal transmission. AIDS Res Hum Retroviruses.

[B6] Ahmad N, Baroudy BM, Baker RC, Chappey C (1995). Genetic analysis of human immunodeficiency virus type 1 envelope V3 region isolates from mothers and infants after perinatal transmission. J Virol.

[B7] Sundaravaradan V, Hahn T, Ahmad N (2005). Conservation of functional domains and limited heterogeneity of HIV-1 reverse transcriptase gene following vertical transmission. Retrovirology.

[B8] Wellensiek BP, Sundaravaradan V, Ramakrishnan R, Ahmad N (2006). Molecular characterization of the HIV-1 gag nucleocapsid gene associated with vertical transmission. Retrovirology.

[B9] Husain M, Hahn T, Yedavalli VR, Ahmad N (2001). Characterization of HIV type 1 tat sequences associated with perinatal transmission. AIDS Res Hum Retroviruses.

[B10] Ramakrishnan R, Husain M, Holzer A, Mehta R, Sundaravaradan V, Ahmad N (2005). Evaluations of HIV type 1 rev gene diversity and functional domains following perinatal transmission. AIDS Res Hum Retroviruses.

[B11] Yedavalli VR, Chappey C, Matala E, Ahmad N (1998). Conservation of an intact vif gene of human immunodeficiency virus type 1 during maternal-fetal transmission. J Virol.

[B12] Yedavalli VR, Chappey C, Ahmad N (1998). Maintenance of an intact human immunodeficiency virus type 1 vpr gene following mother-to-infant transmission. J Virol.

[B13] Yedavalli VR, Husain M, Horodner A, Ahmad N (2001). Molecular characterization of HIV type 1 vpu genes from mothers and infants after perinatal transmission. AIDS Res Hum Retroviruses.

[B14] Hahn T, Ramakrishnan R, Ahmad N (2003). Evaluation of genetic diversity of human immunodeficiency virus type 1 NEF gene associated with vertical transmission. JBiomed Sci.

[B15] Matala E, Crandall KA, Baker RC, Ahmad N (2000). Limited heterogeneity of HIV type 1 in infected mothers correlates with lack of vertical transmission. AIDS Res Hum Retroviruses.

[B16] Yedavalli VR, Ahmad N (2001). Low conservation of functional domains of HIV type 1 vif and vpr genes in infected mothers correlates with lack of vertical transmission. AIDS Res Hum Retroviruses.

[B17] Hahn T, Ahmad N (2001). Genetic characterization of HIV type 1 gag p17 matrix genes in isolates from infected mothers lacking perinatal transmission. AIDS Res Hum Retroviruses.

[B18] Chan DC, Fass D, Berger JM, Kim PS (1997). Core structure of gp41 from the HIV envelope glycoprotein. Cell.

[B19] Boge M, Wyss S, Bonifacino JS, Thali M (1998). A membrane-proximal tyrosine-based signal mediates internalization of the HIV-1 envelope glycoprotein via interaction with the AP-2 clathrin adaptor. J Biol Chem.

[B20] Day JR, Munk C, Guatelli JC (2004). The membrane-proximal tyrosine-based sorting signal of human immunodeficiency virus type 1 gp41 is required for optimal viral infectivity. J Virol.

[B21] Tencza SB, Miller MA, Islam K, Mietzner TA, Montelaro RC (1995). Effect of amino acid substitutions on calmodulin binding and cytolytic properties of the LLP-1 peptide segment of human immunodeficiency virus type 1 transmembrane protein. J Virol.

[B22] Perrin C, Fenouillet E, Jones IM (1998). Role of gp41 glycosylation sites in the biological activity of human immunodeficiency virus type 1 envelope glycoprotein. Virology.

[B23] Johnson WE, Sauvron JM, Desrosiers RC (2001). Conserved, N-linked carbohydrates of human immunodeficiency virus type 1 gp41 are largely dispensable for viral replication. J Virol.

[B24] Swofford DI (1999). PAUP* Phylogenetic analysis using PArsimony and other methods vol 40.

[B25] Koulinska IN, Chaplin B, Mwakagile D, Essex M, Renjifo B (2003). Hypermutation of HIV type 1 genomes isolated from infants soon after vertical infection. AIDS Res Hum Retroviruses.

[B26] Kuhner MK, Yamato J, Felsenstein J (1995). Estimating effective population size and mutation rate from sequence data using Metropolis-Hastings sampling. Genetics.

[B27] Kuhner MK, Yamato J, Felsenstein J (1998). Maximum likelihood estimation of population growth rates based on the coalescent. Genetics.

[B28] Nielsen R, Yang Z (1998). Likelihood models for detecting positively selected amino acid sites and applications to the HIV-1 envelope gene. Genetics.

[B29] Freed EO, Myers DJ, Risser R (1990). Characterization of the fusion domain of the human immunodeficiency virus type 1 envelope glycoprotein gp41. Proc Natl Acad Sci USA.

[B30] Chen SS, Lee CN, Lee WR, Mclntosh K, Lee TH (1993). Mutational analysis of the leucine zipper-like motif of the human immunodeficiency virus type 1 envelope transmembrane glycoprotein. J Virol.

[B31] Dubay JW, Roberts SJ, Brody B, Hunter E (1992). Mutations in the leucine zipper of the human immunodeficiency virus type 1 transmembrane glycoprotein affect fusion and infectivity. J Virol.

[B32] Wild C, Dubay JW, Greenwell T, Baird T, Oas TG, McDanal C, Hunter E, Matthews T (1994). Propensity for a leucine zipper-like domain of human immunodeficiency virus type 1 gp41 to form oligomers correlates with a role in virus-induced fusion rather than assembly of the glycoprotein complex. Proc Natl Acad Sci USA.

[B33] Weng Y, Yang Z, Weiss CD (2000). Structure-function studies of the self-assembly domain of the human immunodeficiency virus type 1 transmembrane protein gp41. J Virol.

[B34] Cheng-Mayer C, Brown A, Harouse J, Luciw PA, Mayer AJ (1999). Selection for neutralization resistance of the simian/human immunodeficiency virus SHIVSF33A variant in vivo by virtue of sequence changes in the extracellular envelope glycoprotein that modify N-linked glycosylation. J Virol.

[B35] Reitter JN, Means RE, Desrosiers RC (1998). A role for carbohydrates in immune evasion in AIDS. Nat Med.

[B36] Ohagen A, Devitt A, Kunstman KJ, Gorry PR, Rose PP, Korber B, Taylor J, Levy R, Murphy RL, Wolinsky SM, Gabuzda D (2003). Genetic and functional analysis of full-length human immunodeficiency virus type 1 env genes derived from brain and blood of patients with AIDS. J Virol.

[B37] Salzwedel K, Johnston PB, Roberts SJ, Dubay JW, Hunter E (1993). Expression and characterization of glycophospholipid-anchored human immunodeficiency virus type 1 envelope glycoproteins. J Virol.

[B38] Weiss CD, White JM (1993). Characterization of stable Chinese hamster ovary cells expressing wild-type, secreted, and glycosylphosphatidylinositol-anchored human immunodeficiency virus type 1 envelope glycoprotein. J Virol.

[B39] Miyauchi K, Komano J, Yokomaku Y, Sugiura W, Yamamoto N, Matsuda Z (2005). Role of the specific amino acid sequence of the membrane-spanning domain of human immunodeficiency virus type 1 in membrane fusion. J Virol.

[B40] Wyss S, Berlioz-Torrent C, Boge M, Blot G, Honing S, Benarous R, Thali M (2001). The highly conserved C-terminal dileucine motif in the cytosolic domain of the human immunodeficiency virus type 1 envelope glycoprotein is critical for its association with the AP-1 clathrin adaptor [correction of adapter]. J Virol.

[B41] Rapoport I, Chen YC, Cupers P, Shoelson SE, Kirchhausen T (1998). Dileucine-based sorting signals bind to the beta chain of AP-1 at a site distinct and regulated differently from the tyrosine-based motif-binding site. Embo J.

[B42] Piller SC, Dubay JW, Derdeyn CA, Hunter E (2000). Mutational analysis of conserved domains within the cytoplasmic tail of gp41 from human immunodeficiency virus type 1: effects on glycoprotein incorporation and infectivity. J Virol.

[B43] Tencza SB, Mietzner TA, Montelaro RC (1997). Calmodulin-binding function of LLP segments from the HIV type 1 transmembrane protein is conserved among natural sequence variants. AIDS Res Hum Retroviruses.

[B44] Kilby JM, Hopkins S, Venetta TM, DiMassimo B, Cloud GA, Lee JY, Alldredge L, Hunter E, Lambert D, Bolognesi D, Mathews T, Johnson MR, Nowak MA, Shaw GM, Saag MS (1998). Potent suppression of HIV-1 replication in humans by T-20, a peptide inhibitor of gp41-mediated virus entry. Nat Med.

[B45] Lu J, Sista P, Giguel F, Greenberg M, Kuritzkes DR (2004). Relative replicative fitness of human immunodeficiency virus type 1 mutants resistant to enfuvirtide (T-20). J Virol.

[B46] Poveda E, Rodes B, Labernardiere JL, Benito JM, Toro C, Gonzalez-Lahoz J, Faudon JL, Clavel F, Schapiro J, Soriano V (2004). Evolution of genotypic and phenotypic resistance to Enfuvirtide in HIV-infected patients experiencing prolonged virologic failure. J Med Virol.

[B47] Heil ML, Decker JM, Sfakianos JN, Shaw GM, Hunter E, Derdeyn CA (2004). Determinants of human immunodeficiency virus type 1 baseline susceptibility to the fusion inhibitors enfuvirtide and T-649 reside outside the peptide interaction site. J Virol.

[B48] Borkow G, Lara HH, Lapidot A (2003). Mutations in gp41 and gp120 of HIV-1 isolates resistant to hexa-arginine neomycin B conjugate. Biochem Biophys Res Commun.

[B49] Ugolini S, Mondor I, Parren PW, Burton DR, Tilley SA, Klasse PJ, Sattentau QJ (1997). Inhibition of virus attachment to CD4+ target cells is a major mechanism of T cell line-adapted HIV-1 neutralization. J Exp Med.

[B50] Stiegler G, Kunert R, Purtscher M, Wolbank S, Voglauer R, Steindl F, Katinger H (2001). A potent cross-clade neutralizing human monoclonal antibody against a novel epitope on gp41 of human immunodeficiency virus type 1. AIDS Res Hum Retroviruses.

[B51] Rowland-Jones SL, Phillips RE, Nixon DF, Gotch FM, Edwards JP, Ogunlesi AO, Elvin JG, Rothbard JA, Bangham CR, Rizza CR, McMichael AJ (1992). Human immunodeficiency virus variants that escape cytotoxic T-cell recognition. AIDS Res Hum Retroviruses.

[B52] Price DA, Goulder PJ, Klenerman P, Sewell AK, Easterbrook PJ, Troop M, Bangham CR, Phillips RE (1997). Positive selection of HIV-1 cytotoxic T lymphocyte escape variants during primary infection. Proc Natl Acad Sci USA.

[B53] Goulder PJ, Brander C, Tang Y, Tremblay C, Colbert RA, Addo MM, Rosenberg ES, Nguyen T, Allen R, Trocha A, Altfeld M, He S, Bunce M, Funchouser R, Pelto SI, Burchett SK, mcIntosh K, Korber BT, Walker BD (2001). Evolution and transmission of stable CTL escape mutations in HIV infection. Nature.

[B54] Geels MJ, Cornelissen M, Schuitemaker H, Anderson K, Kwa D, Maas J, Dekker JT, Baan E, Zorgdrager F, van den Burg R, van Beelen M, Lukashov VV, Fu TM, Paxton WA, van der Hoek L, Dubey SA, Shiver JW, Goudsmit L (2003). Identification of sequential viral escape mutants associated with altered T-cell responses in a human immunodeficiency virus type 1-infected individual. J Virol.

[B55] Wilson CC, Brown RC, Korber BT, Wilkes BM, Ruhl DJ, Sakamoto D, Kunstman K, Luzuriaga K, Hanson IC, Widmayer SM, Wiznia A, Clapp S, Ammann AJ, Koup RA, Wolinsky SM, Walker BD (1999). Frequent detection of escape from cytotoxic T-lymphocyte recognition in perinatal human immunodeficiency virus (HIV) type 1 transmission: the ariel project for the prevention of transmission of HIV from mother to infant. J Virol.

[B56] Hunter E, Korber BHB, Foley B, Mellors J, Leitner T, Myers G, McCutchan F, Kuiken C (1997). 5gp41, a multifunctional protein involved in HIV entry and pathogenesis. Human Retrovirus and AIDS.

[B57] Lu M, Stoller MO, Wang S, Liu J, Fagan MB, Nunberg JH (2001). Structural and functional analysis of interhelical interactions in the human immunodeficiency virus type 1 gp41 envelope glycoprotein by alanine-scanning mutagenesis. J Virol.

[B58] Epstein LG, Sharer LR (1994). Neurological manifestations of perinatally acquired HIV-1 infection. Semin Pediatr Neurol.

[B59] Kao SM, Miller ED, Su L (2001). A leucine zipper motif in the cytoplasmic domain of gp41 is required for HIV-1 replication and pathogenesis in vivo. Virology.

[B60] Labrosse B, Labernardiere JL, Dam E, Trouplin V, Skrabal K, Clavel F, Mammano F (2003). Baseline susceptibility of primary human immunodeficiency virus type 1 to entry inhibitors. J Virol.

[B61] Derdeyn CA, Decker JM, Sfakianos JN, Wu X, O'Brien WA, Ratner L, Kappes JC, Shaw GM, Hunter E (2000). Sensitivity of human immunodeficiency virus type 1 to the fusion inhibitor T-20 is modulated by coreceptor specificity defined by the V3 loop of gp120. J Virol.

[B62] Reeves JD, Gallo SA, Ahmad N, Miamidian JL, Harvey PE, Sharron M, Pohlmann S, Sfakianos JN, Derdeyn CA, Blumenthal R, Hunter E (2002). Sensitivity of HIV-1 to entry inhibitors correlates with envelope/coreceptor affinity, receptor density, and fusion kinetics. Proc Natl Acad Sci USA.

[B63] Matala E, Hahn T, Yedavalli VR, Ahmad N (2001). Biological characterization of HIV type 1 envelope V3 regions from mothers and infants associated with perinatal transmission. AIDS Res Hum Retroviruses.

[B64] Wolinsky SM, Wike CM, Korber BT, Hutto C, Parks WP, Rosenblum LL, Kunstman KJ, Furtado MR, Munoz JL (1992). Selective transmission of human immunodeficiency virus type-1 variants from mothers to infants. Science.

[B65] (1987). Classification system for human immunodeficiency virus (HIV) infection in children under 13 years of age. MMWR Morb Mortal Wkly Rep.

[B66] Adachi A, Gendelman HE, Koenig S, Folks T, Willey R, Rabson A, Martin MA (1986). Production of acquired immunodeficiency syndrome-associated retrovirus in human and nonhuman cells transfected with an infectious molecular clone. J Virol.

[B67] Chenna R, Sugawara H, Koike T, Lopez R, Gibson TJ, Higgins DG, Thompson JD (2003). Multiple sequence alignment with the Clustal series of programs. Nucleic Acids Res.

[B68] Posada D, Crandall KA (2001). Selecting models of nucleotide substitution: an application to human immunodeficiency virus 1 (HIV-1). Mol Biol Evol.

[B69] Posada D, Crandall KA (1998). MODELTEST: testing the model of DNA substitution. Bioinformatics.

[B70] Akaike H (1974). A new look at the statistical model identification. IEEE Trans Autom Contrib.

[B71] Fetching the COALESCE program. http://evolution.genetics.washington.edu/lamarc/coalesce.html.

[B72] Phylogentic Analysis by Maximum Likelihood (PAML), version 3.0. http://abacus/gene.ucl.ac.uk.software/paml.html.

